# Antibiotic Prophylaxis in Orthopaedic Surgery: A Review and Institutional Experience

**DOI:** 10.7759/cureus.87385

**Published:** 2025-07-06

**Authors:** Hamza Ahmed, Farah Mazhar, Aima Gilani, Numan Shah, Aarish Azeem, Marium Rizwan, Abdur Rehman

**Affiliations:** 1 Trauma and Orthopaedics, Salford Royal National Health Service (NHS) Foundation Trust, Salford, GBR; 2 Spinal Surgery, Salford Royal National Health Service (NHS) Foundation Trust, Salford, GBR

**Keywords:** bone and joint infections, orthopaedics infection, orthopaedics surgery, prophylactic antibiotics, surgical site infections (ssi)

## Abstract

Background: Surgical site infections (SSIs) are a significant complication in orthopaedic surgery. Effective use of antimicrobial prophylaxis is critical in reducing their incidence without promoting resistance.

Objectives: To review current evidence on antibiotic prophylaxis in orthopaedic surgery and to analyse data from our institution on the effectiveness of current prophylaxis protocols.

Methods: We carried out a literature review and a retrospective analysis of 1,000 orthopaedic surgeries done over a two-year period. The timing and duration of antibiotic prophylaxis and its association with postoperative SSI rates were evaluated. The standard was compared with the current antibiotic prophylaxis guidelines from our trust policy.

Results: Our institutional data demonstrated no statistically significant difference in SSI rates between patients who received 24-hour versus those who received extended antibiotic prophylaxis. Recent literature consistently supports the timely administration of preoperative antibiotics and discourages prolonged use beyond 24 hours. This is in line with our trust policy.

Conclusion: Preoperative administration of a single dose of antibiotic or a maximum of two doses remains effective in preventing SSIs. Extending prophylaxis beyond 24 hours offers no additional benefit and may promote antimicrobial resistance.

## Introduction

Surgical site infections (SSIs) continue to be a prominent cause of postoperative morbidity in orthopaedic surgery. These infections result in prolonged hospitalisations, additional surgical interventions, and increased healthcare costs. Antimicrobials play a critical role in mitigating this risk, accounting for up to 20% of hospital pharmacy budgets [1].

However, indiscriminate and extended antibiotic use contributes to the alarming rise in antimicrobial resistance (AMR), one of the foremost public health challenges globally. In addition, prolonged antibiotic use is associated with adverse events such as gastrointestinal disturbances and *Clostridium difficile* infections [2].

Surgical antimicrobial prophylaxis aims not to sterilise tissues but to reduce intraoperative bacterial contamination. To be effective, the chosen antimicrobial must be administered at the correct time to ensure optimal tissue concentrations during surgery and be active against the most likely pathogens [3]. Guidelines emphasise the importance of selecting agents that are cost-effective, safe, and have minimal impact on the patient's normal flora [4].

Despite well-established guidelines, significant variation exists in clinical practice regarding the duration and choice of antibiotic prophylaxis. This study aims to both review the existing literature on antimicrobial prophylaxis in orthopaedic surgery and analyse our institutional data to assess the effectiveness of current practices.

## Materials and methods

Study design and setting

A retrospective observational study was conducted at a tertiary care institution. Data was analysed retrospectively from all patients who underwent orthopaedic surgical procedures between April 27, 2023, and April 27, 2025. 

Patient population

The study included 1,000 consecutive patients undergoing emergency or elective orthopaedic surgery. Inclusion criteria were age >18 years and receipt of antibiotic prophylaxis. Exclusion criteria included revision surgeries for infection, immunocompromised status, or incomplete documentation.

Data collection

Data extracted from electronic medical records included demographics such as age and sex, the type of surgery (elective versus emergency), the type and timing of antibiotic administration, and the duration of antibiotic prophylaxis (categorised as a single dose, 24 hours, or more than 24 hours). Additional information collected included the presence of drains or catheters and postoperative complications, specifically surgical site infections (SSI).

SSIs were defined based on Centers for Disease Control and Prevention (CDC) criteria, including infection at the surgical site within 30 days of the procedure or 90 days if an implant was used.

Statistical analysis

Descriptive statistics were used to summarise patient demographics and infection rates. Chi-square tests were used to compare infection rates between groups. A p-value of <0.05 was considered statistically significant.

## Results

Patient characteristics and outcomes

Of the 1,000 patients, 600 (60%) underwent elective surgery, while 400 (40%) underwent emergency surgery. The mean age was 57.4 years, with a range from 19 to 89 years. Of the total, 540 patients were male and 460 were female.

Table 1 summarises infection rates based on the duration of prophylaxis.

**Table 1:** Surgical site infection rates based on prophylaxis duration Data shown as N (%). A chi-square test was used. Statistical significance threshold: p<0.05.

Prophylaxis Duration	No. of Patients (N)	SSI Cases (n, %)	Test Statistic (χ²)	p-value
Single preoperative dose	312	3 (0.96%)		
24-hour prophylaxis	422	3 (0.71%)	χ² = 0.19	0.91
>24-hour prophylaxis	266	2 (0.75%)		
Total	1,000	8 (0.8%)		

There was no statistically significant difference in SSI rates between the three groups (p = 0.91).

Table 2 shows the comparison between elective patients and trauma patients. 

**Table 2:** SSI incidence by surgery type Data shown as N (%). A chi-square test was used. Significance set at p<0.05. SSI: Surgical site infections

Surgery Type	Patients (N)	SSIs (n, %)	Test Statistic (χ²)	p-value
Elective	600	4 (0.67%)		
Emergency	400	4 (1.0%)	χ² = 0.40	0.53

## Discussion

Our study findings corroborate multiple previous studies indicating that extending antibiotic prophylaxis beyond 24 hours does not significantly reduce the incidence of SSIs.

Timing matters

As demonstrated by Classen et al., administering antibiotics within two hours before surgical incision is associated with the lowest rates of SSI [3]. Delayed administration, even by a few hours, increases infection risk substantially.

Single vs. multiple doses

Numerous randomised trials and meta-analyses support the finding that single-dose or 24-hour regimens are as effective as prolonged courses [5-8]. The Dutch Trauma Trial [7], for example, showed a significant reduction in SSIs in patients receiving a preoperative dose of ceftriaxone compared to placebo.

Impact of extended use

Prolonged antibiotic use does not offer additional protection, but it has been shown to increase the risk of AMR, adverse reactions, and healthcare costs [9-11]. Gillespie and Walenkamp’s meta-analysis involving 8,307 patients showed no advantage of multi-dose over single-dose prophylaxis in preventing infections related to closed fracture fixation [4].

Institutional insights

In our cohort of 1,000 patients, we observed low infection rates across all groups that were quite similar, with no statistical benefit from extending antibiotic prophylaxis. Our findings align closely with the broader literature and current evidence-based guidelines, and our trust policy is compliant with them.

Trust policy

Our trust policy is summarised in Tables 3, 4. This is the policy of the Northern Care Alliance - Salford Care Organisation, Manchester, United Kingdom. 

**Table 3:** Antimicrobial prophylaxis guidelines for orthopaedic surgery Information taken from the local trust policy at Salford Care Organisation.

Procedure Type	1st Line Prophylaxis	Regimen for Penicillin Allergy	If MRSA Cover Needed
Closed clean orthopaedic procedures without prosthesis/implants (e.g., arthroscopy)	No prophylaxis recommended	No prophylaxis recommended	No prophylaxis recommended
Joint replacement (hip, knee, shoulder, elbow, ankle)	Flucloxacillin 2g IV (1g if GFR ≤10) + Gentamicin IV at induction. Followed by Flucloxacillin 1g IV at 6, 12, 18 hours post-induction. If surgery >3 hours, give an extra 1g intra-op	Teicoplanin IV + Gentamicin IV at induction	Teicoplanin IV + Gentamicin IV at induction
Prosthetic joint revision	Teicoplanin IV + Gentamicin IV at induction	Same as MRSA regimen	Same as penicillin allergy regimen
Other orthopaedic procedures requiring prophylaxis (e.g., internal fixation, day case hand surgery)	Flucloxacillin 2g IV (1g if GFR ≤10) + Gentamicin IV at induction. No further post-op doses	Teicoplanin IV + Gentamicin IV at induction	Teicoplanin IV + Gentamicin IV at induction

**Table 4:** Prophylaxis for open fractures Information taken from the local trust policy at Salford Care Organisation.

Open Fracture Type/Stage	1st Line	Penicillin Allergy	MRSA Risk
Initial management (within 3 hours of injury)	IV Co-amoxiclav 1.2g TDS (24 hours only)	IV Clindamycin 600mg QDS (24 hours only)	IV Teicoplanin + IV Metronidazole 500mg TDS (24 hours only)
Gustilo Grade 1 - At induction for first debridement	IV Co-amoxiclav + single dose IV Gentamicin	IV Clindamycin + single dose IV Gentamicin	IV Teicoplanin 800mg + IV Gentamicin
Gustilo Grade 2 & 3 - At induction for first debridement	Continue Co-amoxiclav (up to 72 hours or until closure) + single dose IV Gentamicin	Continue Clindamycin (up to 72 hours or until closure) + single dose IV Gentamicin	Teicoplanin 12-hourly ×3 then daily + Metronidazole 500mg TDS (up to 72 hours or until closure)
At skeletal stabilisation & definitive soft tissue closure	IV Co-amoxiclav 1.2g (if >24 hours since last dose) + single dose IV Gentamicin	IV Clindamycin 600mg + single dose IV Gentamicin	IV Teicoplanin 800mg + IV Gentamicin (max 800mg if already on teicoplanin)

Antimicrobial prophylaxis is recommended for certain clean orthopaedic procedures and surgeries involving non-sterile mucosal surfaces, following local departmental protocols and recorded in the EPMAR. Teicoplanin should be included in the regimen for patients at high risk of MRSA infection, such as those with a history of MRSA colonisation, recent hospitalisation, or from high-risk environments.

Prophylactic antibiotics must be administered within 60 minutes before surgical incision and before tourniquet inflation if used. Flucloxacillin should be given as a slow IV injection over 3-4 minutes, and gentamicin as a 20-minute infusion. Care must be taken during induction to prevent medication errors that could lead to intraoperative awareness.

Prophylactic regimens vary by procedure type, with special considerations for penicillin-allergic patients and those requiring MRSA coverage. Open fractures require staged antimicrobial management based on Gustilo classification and timing of debridement or closure.

Limitations

This study was retrospective in nature, and reliance was mostly on recorded data. There is also a chance of potential under-reporting of late-presenting SSIs. In addition to this, there is an absence of stratification by comorbidities or type of implant. We recommend that future prospective randomised trials, ideally stratified by fracture type, comorbid status, and surgical complexity, would help refine prophylactic protocols further.

## Conclusions

Antibiotic prophylaxis remains a cornerstone in preventing SSIs in orthopaedic surgery. Current evidence strongly supports the use of a single or two doses of preoperative antibiotics within 24 hours. Extending prophylaxis beyond 24 hours confers no additional benefit and contributes to antibiotic resistance and other complications. Institutional practices should be aligned with international guidelines to optimise patient outcomes while minimising unnecessary antimicrobial exposure.
